# Evaluating predictive screening for children's post-injury mental health: New data and a replication

**DOI:** 10.3402/ejpt.v6.29313

**Published:** 2015-12-14

**Authors:** Nancy Kassam-Adams, Meghan L. Marsac, J. Felipe García-España, Flaura Winston

**Affiliations:** 1 Center for Injury Research and Prevention, Children's Hospital of Philadelphia, Philadelphia, PA, USA; 2 Department of Pediatrics, Perelman School of Medicine, University of Pennsylvania, Philadelphia, PA, USA; 3 Department of Child and Adolescent Psychiatry, Perelman School of Medicine, University of Pennsylvania, Philadelphia, PA, USA; 4 Children's Hospital of Philadelphia Research Institute, Philadelphia, PA, USA

**Keywords:** screening tools, injury, PTSD, depression, PTSD prediction, stepped care

## Abstract

**Background:**

Recommended approaches for secondary prevention of posttrauma mental health difficulties in children require empirically sound predictive screening to determine which children require more intensive monitoring or targeted intervention. Although there are several promising screening tools for injured children, none has emerged as the gold standard, and little replication data are available regarding their performance.

**Objective:**

To evaluate a predictive screening protocol for risk of later posttraumatic stress (PTS) and depression outcomes and address a crucial lack of replication studies by examining performance of two previously published screening tools (Screening Tool for Early Predictors of PTSD [STEPP] and Child Trauma Screening Questionnaire [CTSQ]).

**Method:**

The study enrolled 290 children hospitalized after acute injury. A three-part screening protocol, including acute PTS and depression symptoms and other empirically derived risk factors, was administered in hospital as part of a stepped care study. PTS and depression symptoms and health-related quality of life (HRQoL) were assessed 6 months post-injury.

**Results:**

The screening protocol demonstrated excellent sensitivity (1.00) and good specificity (0.73) for prediction of 6-month PTS, moderate sensitivity (0.64) and good specificity (0.74) for 6-month depression, and excellent negative predictive value for both outcomes. Among children screening at risk, HRQoL was poorer at 6 months post-injury. Replication analyses found predictive utility (sensitivity and specificity) was low for the STEPP and moderate for the CTSQ.

**Conclusions:**

This study provides additional evidence that early post-injury screening could identify children at higher risk for persistent PTS symptoms and limited support for predicting post-injury depression. Findings support acute PTS symptoms as key early risk markers. The predictive value of a negative screening result (i.e., knowing who is not at risk) may be especially important in choosing where to target limited follow-up resources. It is crucial that future investigations provide additional replication data regarding existing screening tools and evaluate additional or alternate items (proposed *a priori*) to improve predictive power.

After experiencing an injury, many children and youth have initial traumatic stress reactions and other disturbances of mood or behavior (Winston et al., 2002). While most recover well, a substantial minority will go on to have persistent posttraumatic mental health concerns—especially posttraumatic stress (PTS) and depression symptoms—and decreased health-related quality of life (HRQoL) (Kassam-Adams, Marsac, Hildenbrand, & Winston, 2013). Recommended frameworks for the secondary prevention of problematic psychological sequelae of injuries and other acute trauma in children suggest “watchful waiting” to identify those most at risk and stepped care models that match level of risk with appropriate levels of intervention (Kassam-Adams, 2014; McDermott & Cobham, 2014; National Institute for Clinical Excellence, 2005). Each of these recommended approaches requires empirically sound predictive screening methods to determine which children require more intensive monitoring or targeted professional intervention.

The potential for brief screening of injured children to predict who is most at risk for poorer psychological outcomes was introduced over a decade ago (Winston, Kassam-Adams, Garcia-Espana, Ittenbach, & Cnaan, 2003), and while several promising screening tools have been created (Kenardy, Spence, & Macleod, 2006; Kramer, Hertli, & Landolt, 2013; Nixon, Ellis, Nehmy, & Ball, 2010; Olsson, Kenardy, De Young, & Spence, 2008; Winston et al., 2003), no instrument has emerged as the gold standard for this purpose. Several published reports have explicitly proposed predictive screening tools for persistent PTS symptoms in injured children and tested the utility (i.e., sensitivity and specificity) of these tools in prospective studies (see Table 1). Although several of these same tools have also been evaluated as predictive tools for persistent post-injury depression, to our knowledge, no investigators have proposed tools designed explicitly to predict risk of post-injury depression in children. In addition to purpose-designed screening tools for PTS risk, there have been several reports regarding the predictive utility of various combinations of acute PTS symptoms as predictors of later PTS or depression symptoms (Dalgleish et al., 2008; Kassam-Adams & Winston, 2004; Meiser-Stedman, Yule, Smith, Glucksman, & Dalgleish, 2005).

**Table 1:** Summary of empirically tested predictive screening tools for persistent PTS or depression symptoms in injured children

			Published studies testing predictive utility	
			
Screening tool	Number of items/item content	Age range	Posttraumatic stress	Depression
STEPP	8 items/traumatic stress reactions and other risk factors	School age Adolescent	(Winston et al., 2003) (Nixon, Ellis, et al., 2010) (Van Meijel et al., 2015)	(Winston et al., 2003) (Nixon, Ellis, et al., 2010)
CTSQ	10 items/traumatic stress reactions, tested with/without heart rate item	School age Adolescent	(Kenardy et al., 2006) & (Olsson et al., 2008) from same sample (Nixon, Ellis, et al., 2010)^a^	None
STEPP-AUS	8 items/traumatic stress reactions and other risk factors	School age Adolescent	(Nixon, Ellis, et al., 2010)	(Nixon, Ellis, et al., 2010)
PEDS-ES	21–26 items/emotional distress reactions and other risk factors	Preschool	(Kramer et al., 2013)	None

a Tested items with similar content. PTS=posttraumatic stress; STEPP=Screening Tool for Early Predictors of PTSD; CTSQ=Child Trauma Screening Questionnaire; STEPP-AUS=Australian version of the STEPP; PEDS-ES=Pediatric Emotional Distress Scale—Early Screener.

The past 15 years have seen the development of a growing empirical literature regarding risk factors and etiological models for the development of persistent PTS symptoms in children after injury (Kassam-Adams et al., 2013), and investigators have begun to examine risk factors for post-injury depression symptoms in children (Han et al., 2011; Pailler, Kassam-Adams, Datner, & Fein, 2007). This literature provides a solid grounding for the development of predictive screening tools, but determining an optimal brief set of predictive items is conceptually distinct from delineating key etiological factors. The set of items that optimize predictive screening for post-injury PTS or depression outcomes may include non-causal risk markers as well as causal etiological factors (Kraemer et al., 1997). Screening tools developed to date have employed early traumatic stress symptoms and/or other empirically derived risk factors as predictive items. No study to date has provided a comprehensive examination, in a single sample, of the relative predictive utility of early symptoms and other risk markers.

## Objective

The current paper has two primary objectives: first, to report on the performance of a predictive screening protocol administered to children within 2 weeks post-injury as part of a stepped care program, and second, to address a crucial lack of replication studies by examining the performance of two previously developed screening tools: the Screening Tool for Early Predictors of PTSD (STEPP) (Winston et al., 2003), which is composed of early risk markers, and the Child Trauma Screening Questionnaire (CTSQ) (Kenardy et al., 2006), which is composed of acute PTS symptoms. The three-component screening protocol designed for the stepped care program aimed to predict risk of psychosocial distress (PTS or depression symptoms) 6 months post-injury by employing (a) empirically derived risk factors, (b) acute PTS symptoms, and (c) acute depression symptoms. We hypothesized that screening positive on any one of these components would prospectively predict poorer posttraumatic mental health outcomes (PTS and depression) at 6 months and would also be associated with lower HRQoL at 6 months (i.e., convergent validity).

Despite a growing reliance on screening to guide early intervention and prevention, there is currently a paucity of evidence regarding the performance of specific screening tools for children. Each predictive screening tool previously proposed in the literature (see Table 1) demonstrated reasonably good predictive utility within the study in which it was developed or first evaluated. However, there have been few attempts to formally evaluate the performance of these tools in new samples of injured children, and there have been only two published reports of such analyses (Nixon, Nehmy, et al., 2010; Van Meijel et al., 2015). Thus the second goal of the current paper is to help address this gap by reporting on the predictive utility of two proposed screening tools, the STEPP (Winston et al., 2003), and a version of the CTSQ (Kenardy et al., 2006).

## Method

The current analyses use data from an overall study that was designed as a pilot randomized trial evaluating a stepped care preventive intervention. Enrollment procedures and eligibility criteria have been described in detail elsewhere (Kassam-Adams et al., 2011). In summary, we enrolled 290 children aged 8–17 years who were admitted to a large urban pediatric hospital in the United States for treatment of an acute unintentional injury. All research procedures were conducted in accordance with an IRB-approved study protocol. In the hospital, always within 2 weeks of injury (mean=2.3 days post-injury), participants completed baseline assessments. The first assessment administered was a three-part risk screening protocol (described below). Follow-up research assessments, conducted 6 weeks and 6 months post-injury by research staff unaware of the child's risk status or treatment assignment, included measures of child PTS symptoms, depression symptoms, and HRQoL. The current analyses address 6-month follow-up outcomes, in order to provide the most rigorous test of this early screening protocol for prediction of longer term outcomes.

**Fig. 1:**
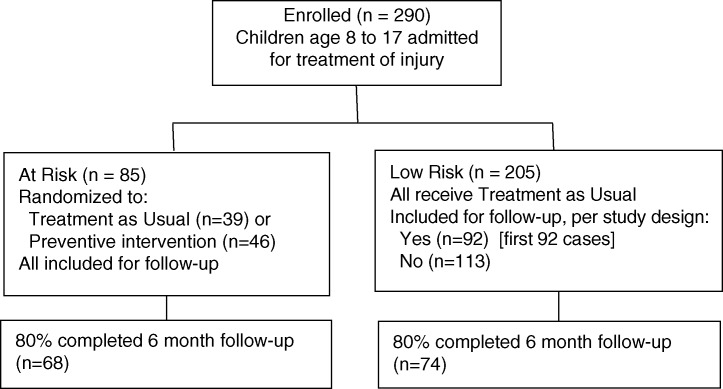
Participant flowchart for study screening protocol.

See Fig. 1 for the flow of participants in this study design. The stepped intervention examined in the pilot RCT required a determination of risk for persistent PTS symptoms, so that only those children determined to be “at risk” would participate in the RCT portion of the study. Thus, for purposes of RCT enrollment, the research team selected screening measures and established a “rule” for determining “at-risk” status based on empirical evidence available at the time the study was initiated. Among enrolled participants, those determined to be “at risk” based on the screening protocol (*N*=85) were randomized to receive a stepped preventive care intervention or usual care. Results of this pilot randomized trial did not reveal any differences in outcomes (PTS and depression symptoms, HRQoL) between treatment conditions (Kassam-Adams et al., 2011), thus these groups are combined in the current analyses. All of these participants were included for follow-up assessments.

### Inclusion of a portion of the “low-risk” participants for follow-up assessment

In a “real-world” application of the stepped care intervention program, those children deemed to be “low risk” would have received no additional formal follow-up from the healthcare team. However, for purposes of the study, a portion of those determined to be “low risk” based on the screening protocol (specifically, the first 92 participants who screened as “low risk”) were included for follow-up assessments of PTS and depression symptoms and HRQoL. (Low-risk participants included versus not included for follow-up did not differ with regard to age, gender, race/ethnicity, circumstances of injury, or baseline PTS or depression symptom severity.) The inclusion of these “low-risk” participants for follow-up allows us to examine the accuracy of the screening protocol's risk determinations, utilizing corrections for partial verification bias (Begg & Greenes, 1983), which are described in detail below.

Thus, of the 290 children enrolled, 177 were eligible for follow-up assessments (85 “at risk” plus 92 “low risk”). Among these 177 children, 122 (69%) were male, mean age was 12.1 years (SD 2.5); 110 (62%) were White, 55 (31%) Black, and 12 (7%) of other ethnicities. The circumstances of injury were recreational activities (51, 29%), organized sports (47, 27%), falls (38, 22%), motor vehicle crashes (27, 15%), animal bites (7, 3%), and other circumstances (7, 4%). Among the 290 children initially enrolled, those eligible versus not eligible for follow-up did not differ with regard to child age, gender, race/ethnicity, or circumstances of injury.

### Measures

Demographic, injury, and treatment information was obtained from parent report and abstracted from the medical record.

### Screening protocol components

The screening protocol consisted of three components (described below). For purposes of the RCT, children were considered to be “at risk” based on this screening protocol if they had a positive screen on one or more of the three components.

Screening component 1: Positive screen on the STEPP (Winston et al., 2003). The STEPP is a brief 8-item screening tool administered during acute trauma care to identify injured children at higher risk for persistent traumatic stress (one item asked of the parent, four items asked of the child, and three items recorded from the child's medical record). The development of the STEPP is described in detail elsewhere (Winston et al., 2003); in brief, in the development sample, STEPP items were empirically selected, from a larger theoretically derived survey of risk factors, to optimize predictive power for persistent child PTS symptoms. The eight STEPP items that predict persistent child traumatic stress include pre-trauma factors (prior behavior problems and female sex) and peri-trauma factors (whether others were hurt/injured in the same event, separation from parents, intense fear, subjective life threat, presence of an extremity fracture injury, and elevated heart rate at emergency department triage). The child STEPP screen is positive when at least four of these eight items are endorsed. In a development sample with injured pediatric patients, the STEPP had excellent sensitivity (0.88), and moderate specificity (0.48) in predicting 6-month PTS (Winston et al., 2003).

Screening component 2: Positive screen (score of 15 or greater) on the Child PTSD Symptom Scale (CPSS) (Foa, Johnson, Feeny, & Treadwell, 2001). The CPSS is a self-report instrument that yields both a PTS symptom severity score and a determination of likely PTSD diagnostic status according to *DSM*-IV symptom criteria. Seventeen CPSS items correspond to the *DSM*-IV symptom criteria and are summed to create the CPSS symptom severity score; seven additional items assess impairment from those symptoms. The CPSS has shown excellent internal consistency, test-retest reliability, and convergent validity with structured clinical interview measures of PTSD (Foa et al., 2001; Kassam-Adams, Marsac, & Cirilli, 2010; Kataoka et al., 2003). The original CPSS psychometric paper (Foa et al., 2001), based on a sample of 75 children assessed 2 years post-earthquake exposure, recommended a cutoff of ≥11 on the CPSS symptom severity score to denote current clinically significant PTS symptoms. In developing the screening protocol for this study of injured children, we examined data from several prior samples (total *N*=370) of injured children to determine an optimal cutoff score. Based on analyses that compared CPSS symptom severity scores to a categorical “diagnosis” scored from the same measure, we determined that a CPSS symptom severity score ≥15 afforded excellent sensitivity (1.00) and specificity (0.90) for the presence of significant PTS symptoms.

Screening component 3: Positive screen (score of 24 or greater) on the Center for Epidemiologic Studies Depression Scale (CES-D) (Radloff, 1977). The CES-D is a 20-item self-report measure of depression symptoms that yields a total severity score. Clinical cutoff scores (≥16 for adults and ≥24 for youth) have been empirically established (Dierker et al., 2001; Houston et al., 2001; Radloff, 1977). The CES-D has been validated in adults and children as young as 10 and over as an effective screen for depression. It has been successfully used in studies with children as young as 8, demonstrating strong psychometric properties (Kassam-Adams, Bakker, Marsac, Fein, & Winston, 2015; Tatar, Kayiran, Saltukoglu, Ozkut, & Emeksiz, 2013).

### Additional measure for replication analyses

The CTSQ is a 10-item screening tool consisting of early PTS symptoms: five reexperiencing and five arousal symptoms (Kenardy et al., 2006). In a development sample with injured pediatric patients, the CTSQ had excellent sensitivity (0.82), and specificity (0.74) in predicting 6 month PTS (Kenardy et al., 2006). The original CTSQ was not included in this study. However, for the current analyses, following Nixon, Ellis et al. (2010), we derived a modified “CPSS-10” scoring based on the 10 CPSS items whose content parallels that of the CTSQ. Consistent with the cutoff scores reported by the CTSQ developers, in the current analyses children screened positive on the CPSS-10 if they endorsed at least five of the 10 symptoms as present.

### Six-month outcomes

The CPSS and CES-D were administered to children approximately 6 months post-injury to assess persistent PTS and depression symptoms respectively. We determined persistent PTS by scoring the CPSS for presence of *DSM*-IV PTSD symptom criteria and determined persistent depression symptoms based on a CES-D score ≥24. These outcomes are based on self-report measures rather than a clinical diagnostic interview and thus should not be interpreted as a definitive finding of a PTSD or depression diagnosis but rather as the presence of persistent symptoms that may be clinically significant.

The Pediatric Quality of Life Inventory (PedsQL) (Varni, Seid, & Rode, 1999) is a well-validated 23-item measure of child HRQoL. Six-month PedsQL total score based on child self-report is utilized in the current analyses. Potential scores range 0–100; higher scores indicate better functional outcomes.

### Data analyses

The overall study design included baseline assessments of all enrolled participants (*N*=290). However, as noted above, for efficiency in the pilot randomized trial, follow-up research assessments were planned for 177 participants, that is, for all 85 participants designated as “at-risk” and for the first 92 designated as “low-risk” based on the screening protocol. (In other words, based on this design, once we had enrolled 92 low-risk participants, the subsequent low-risk participants (*N*=113) were not followed.) For the current purpose of examining the performance of the screening protocol, this design could lead to “partial verification bias,” which may overestimate sensitivity and underestimate specificity (Begg & Greenes, 1983). Our analytical approach thus needed to correct for this potential bias when examining screener performance. Based on simulation studies comparing several methods for correcting partial verification bias (DeGroot et al., 2011), the method suggested by Begg and Greenes (1983) has been found to be optimal when a study design includes follow-up, that is, “verification,” for a proportion of enrolled participants based on the predictive test score (as in the current study). In this approach, bias is corrected by utilizing observed proportions of outcomes among participants with follow-up (“verified participants”) to calculate the expected number of participants with and without each outcome among those without follow-up (“non-verified participants”). We used this method to correct for partial verification bias, and we report adjusted sensitivity and specificity figures. Positive predictive value (PPV) and negative predictive value (NPV) are not affected and thus not adjusted. Following the same procedures to correct for partial verification bias, we also calculated adjusted sensitivity, adjusted specificity, PPV, and NPV for each component of the screening protocol separately and for the modified CPSS-10 scoring of the CPSS (i.e., paralleling the CTSQ).

We further assessed the convergent validity of the screening protocol in predicting clinically meaningful differences in child well-being 6 months post-injury by examining 6-month HRQoL. We used t-tests to examine mean differences in 6-month total PedsQL score among those who were screened as at-risk versus low-risk based on the study screening protocol, and calculated the effect size (Cohen's d) as the standard mean difference—the between-group mean difference in PedsQL score standardized by the pooled standard deviation of the two groups.

## Results

### Screening protocol and outcome measures

**Fig. 2:**
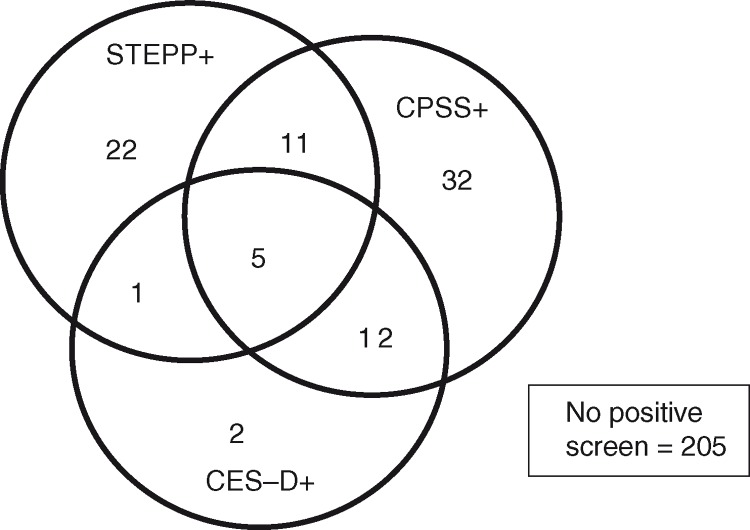
Overlap among positive screening results across the three components of the screening protocol.

Among the 290 children screened, 85 (29%) screened as “at risk” on the overall screening protocol (positive screen on at least one of the three components: STEPP, CPSS, or CES-D); 39 (13%) screened positive on the STEPP, 60 (21%) screened positive on the CPSS, and 20 (7%) screened positive on the CES-D. Figure 2 depicts overlap among positive results on components of the screening protocol. In the CPSS-10 (items similar to the CTSQ screening tool), 32 (11%) children in this sample screened positive with 5 or more symptoms present.

At 6 months post-injury, 142 children (80% of the 177 with whom we intended to follow-up) completed follow-up assessments (113 low-risk participants were intentionally not followed up, as described above). Among these 142 children, 7 (2%) reported symptoms consistent with *DSM*-IV PTSD symptom criteria, 15 (5%) had significant depression symptoms, and mean scores on the PedsQL were 82.6 (SD 18.2) with a range from 2.2 to 100.

### Predictive performance of screening protocol

Table 2 and 3 show the performance of the screening protocol in predicting 6-month PTS and depression outcomes. The three-component screening protocol evidenced perfect sensitivity and very good specificity in predicting 6-month PTS outcomes. The protocol also evidenced perfect NPV. In other words, no child who screened negative on this protocol went on to have persistent PTS symptoms consistent with *DSM*-IV diagnostic criteria. For depression outcomes, the three-component screening protocol showed moderate sensitivity and specificity and nearly perfect NPV.

**Table 2:** Predicting 6-month PTS symptoms (meeting symptom criteria on the CPSS) based on screening administered soon after injury

	Adjusted sensitivity	Adjusted specificity	PPV	NPV
Study screening protocol (any component positive)	1.00	0.73	0.10	1.00
Components of study screener				
STEPP positive	0.16	0.87	0.06	0.95
CPSS positive	1.00	0.82	0.15	1.00
CES-D positive	0.39	0.94	0.21	0.98
CPSS-10 (parallel items to CTSQ)	0.57	0.91	0.20	0.98

STEPP=Screening Tool for Early Predictors of PTSD; CPSS=Child PTSD Symptom Scale; CES-D=Center for Epidemiologic Studies Depression Scale; CTSQ=Child Trauma Screening Questionnaire.

**Table 3:** Predicting 6-month depression symptoms (≥24 on the CESD) based on screening administered soon after injury

	Adjusted sensitivity	Adjusted specificity	PPV	NPV
Study screening protocol (any component positive)	0.64	0.74	0.18	0.96
Components of study screener				
STEPP positive	0.30	0.88	0.21	0.93
CPSS positive	0.44	0.82	0.19	0.94
CES-D positive	0.30	0.95	0.37	0.93
CPSS-10 (parallel items to CTSQ)	0.34	0.91	0.28	0.93

STEPP=Screening Tool for Early Predictors of PTSD; CPSS=Child PTSD Symptom Scale; CES-D=Center for Epidemiologic Studies Depression Scale; CTSQ=Child Trauma Screening Questionnaire.

Table 2 and 3 also present the results of analyses assessing the performance of each component of the protocol and of the CPSS-10. (STEPP and CPSS-10 results can be considered as replication analyses for previously developed screening tools.) Examination of the performance of the separate components indicates that the predictive utility of the protocol for later PTS outcomes derives primarily from the CPSS. The separate components’ performance in predicting 6-month depression is not as clear cut as for PTS outcomes; all components had excellent specificity and NPV. The CPSS-10 (representing items similar to the CTSQ measure) demonstrated only modest sensitivity but strong specificity and NPV in predicting 6-month PTS outcomes. The CTSQ (CPSS-10) was not designed to predict depression outcomes; it showed excellent specificity and NPV but poor sensitivity in predicting 6-month depression symptoms.

### Convergent validity of screening protocol

For HRQoL outcomes, Table 4 shows 6-month PedsQL scores for those screening at-risk versus low-risk, based on the study screening protocol and each of its three components. On average, children screening as “at risk” on the three-component study screening protocol were approximately two-thirds of a standard deviation lower in PedsQL score at 6 months than those who screened as low risk, representing a medium-to-large effect size. Examining each screening component separately, the STEPP screen resulted in a medium effect size, and both the CPSS screen and the CESD screen resulted in large effect sizes for 6-month PedsQL score.

**Table 4:** Six-month health-related quality of life (PedsQL total score) for those screening at-risk versus low-risk soon after injury: mean differences and effect size

	Low risk, *M* (SD)	At-risk, *M* (SD)	*t*, df	*p*	*d*
Study screener (any component positive)	88.2 (11.5)	76.5 (21.9)	3.91, 139	<0.001	0.68
Components of study screener					
STEPP positive	84.6 (16.1)	76.1 (22.8)	2.00, 139	0.05	0.48
CPSS positive	87.5 (12.1)	72.5 (23.8)	4.03, 139	<0.001	0.89
CES-D positive	85.0 (15.1)	67.1 (27.1)	2.82, 139	0.01	1.04

PedsQL=Pediatric Quality of Life Inventory; STEPP=Screening Tool for Early Predictors of PTSD; CPSS=Child PTSD Symptom Scale; CES-D=Center for Epidemiologic Studies Depression Scale; *d*=Standard mean difference in PedsQL total score between groups at 6 months.

## Discussion

A three-component screening protocol, administered soon after unintentional injury (during hospital admission), was extremely successful in identifying children who were at higher risk for persistent PTS symptoms, as well as those who would not go on to have PTS symptoms at 6 months. The screening protocol was somewhat less successful in predicting development of 6-month depression symptoms but performed extremely well in identifying a group of children who would not have ongoing depression at 6 months. The predictive value of a negative screening result (i.e., NPV) may be especially important in a system with limited resources. The ability to identify some injured children who are very unlikely to develop persistent PTS or depression symptoms, and thus do not require additional preventive interventions, could be of great practical value. In terms of PTS outcomes, this screening protocol was highly sensitive (i.e., did not miss any children at risk), but that does not imply that all children who screened positive went on to develop problematic outcomes (just 10% did so). The most appropriate outcome of a positive early screen is likely to be continued follow-up and monitoring. Such monitoring does not need to be labor-intensive and costly. For example, follow-up for PTS or depression symptoms may be integrated within medical follow-up for injury that is routinely carried out by a trauma surgery clinic or the child's general practitioner, or may be delivered online via evidence-based self-directed tools for parents from websites such as www.AfterTheInjury.org or www.kidtrauma.com.

Prior research has not provided clear evidence for the utility of including early PTS or depression symptoms, instead of or in addition to other risk markers, for prediction of longer term psychological distress after injury. For example, several studies have found that a diagnosis of (*DSM*-IV-defined) acute stress disorder is not an optimal predictor of later PTSD diagnosis, yet various combinations of acute PTS symptoms have been seen to be useful in predicting PTSD outcomes (Dalgleish et al., 2008; Kassam-Adams & Winston, 2004; Meiser-Stedman et al., 2005). By evaluating a three-component screening protocol, which included different types of predictive tools, the current study design allowed us to explore the relative predictive utility of early symptoms and other risk factors. Most of the value of this protocol for prediction of PTS outcomes appears to derive from assessment of early traumatic stress symptoms, lending support to the concept of including early PTS symptoms as key early risk markers in screening protocols. In contrast, early depression symptoms did not emerge as clear predictors of depression outcomes. Taking a broader perspective, HRQoL was poorer among children who screened positive at baseline on measures of PTS or depression symptoms. This suggests convergent validity for these early symptoms as markers of risk for less-than-optimal injury recovery, even when recovery is defined more broadly than psychological symptoms.

We also sought to provide crucial data regarding replication of past screening results by examining the performance of two predictive screening tools that achieved promising results in earlier studies. To our knowledge, in the decade since the development of these tools only two other published studies have examined either tool's predictive performance in independent samples (Nixon, Nehmy, et al., 2010; Van Meijel et al., 2015). In one replication study, the STEPP evidenced poor sensitivity (0.22 and 0.25) and moderate specificity (0.67 and 0.67) to predict 6-month PTSD and depression, respectively (Nixon, Ellis, et al., 2010), and in the same sample, the CPSS-10 (CTSQ analog with score ≥5) had moderate sensitivity (0.67) and specificity (0.43) for prediction of 6-month PTSD (Reginald Nixon, personal communication, February 2015). In a second replication study, the STEPP (in Dutch) evidenced only modest sensitivity (0.41) and strong specificity (0.87) with its original scoring; lowering the cutoff score to three items being endorsed yielded moderate sensitivity (0.65) and specificity (0.67) (Van Meijel et al., 2015). Thus, taken together, our current results and results of the only other studies that are currently available (Nixon, Nehmy, et al., 2010; Van Meijel et al., 2015) indicate poor to modest replication (STEPP) or partial replication (CTSQ) of the original predictive performance of these screening tools.

It may not be surprising to find that sensitivity and specificity are somewhat lower in new independent samples compared with a development sample, analogous to finding somewhat attenuated effect sizes when experimental or intervention findings are replicated. With the current analyses, we now have three replication studies examining the STEPP and two examining the CTSQ. These studies were conducted in three countries but in very similar samples of recently injured children recruited in medical settings. For the CTSQ, replication findings are based on CTSQ item content and not exact item wording, and it is possible that CTSQ replication results would be stronger if the original CTSQ item wording had been included in the Nixon et al. study or in the current study. For PTSD prediction in particular, the evidence to date appears strongest for the predictive performance of early PTS symptoms (e.g., on the CPSS or CTSQ), but mixed replication results for the STEPP suggest that it would be premature to rule out other risk markers as predictive tools (Kassam-Adams et al., 2013). Evidence to date does not support the use of the current STEPP tool specifically; more studies would be needed to support the use of additional or alternate items or cutoff scores for the STEPP. The field would benefit from future investigations that explicitly test the predictive utility of promising existing tools, in injured children and in other children exposed to acute trauma, and that evaluate the ability of additional or alternate items (proposed *a priori*) to improve predictive power.

Strengths of the current study include testing a screening protocol derived from past research in a new sample and examining the ability of this protocol to predict more than one post-injury mental health outcome as well as HRQoL. However, there are several limitations of the current study that deserve attention. First, as our study design did not follow all children who were hypothesized to be low-risk, PPV and NPV cannot be used as population estimates (Begg & Greenes, 1983). Next, it is possible that despite the lack of significant findings from the intervention trial in the same sample, there were some intervention effects on the prediction of post-injury mental health outcomes. Future studies that build on and replicate these results are needed. Our use of self-report measures to assess PTS and depression outcomes is a limitation; in that, we cannot conclusively determine PTSD or depression diagnoses, but we believe that these validated self-report measures represent meaningful information regarding child outcomes at 6 months. Lastly, the multicomponent screening protocol used in the current study included 45 items across three measures and would likely be too lengthy for routine use in a busy pediatric medical setting. Future studies should build on the current exploratory results regarding each separate screening component, striving to balance brevity with predictive ability and should explore alternative mechanisms (e.g., electronically delivered self-report screening tools) to efficiently collect screening information from trauma-exposed children.

## Conclusions

Early PTS symptoms, and to a lesser extent early depression symptoms, appear to signal higher risk for poorer posttraumatic mental health and HRQoL after injury. A focus on early PTS symptoms may assist in identifying not only those children with severe early distress who need immediate intervention, but also those who would benefit from ongoing monitoring. Not all children with early PTS symptoms will go on to have persistent distress; thus an appropriate clinical response may follow the model of “watchful waiting” (National Institute for Clinical Excellence, 2005), incorporating ongoing monitoring of child emotional recovery at regular intervals over several months post-injury. Toward the end, future research should address the development of widely accessible and low-cost monitoring and preventive interventions that are feasible for implementation within pediatric healthcare systems and/or via mobile or online access.

## Conflict of interest and funding

This study was funded by a grant from the US Centers for Disease Control and Prevention (R49CE987).
